# Applying Generative Artificial Intelligence to cognitive models of decision making

**DOI:** 10.3389/fpsyg.2024.1387948

**Published:** 2024-05-03

**Authors:** Tyler Malloy, Cleotilde Gonzalez

**Affiliations:** Dynamic Decision Making Laboratory, Department of Social and Decision Sciences, Dietrich College, Carnegie Mellon University, Pittsburgh, PA, United States

**Keywords:** cognitive modeling, decision making, generative AI, instance based learning, natural language, visual learning

## Abstract

**Introduction:**

Generative Artificial Intelligence has made significant impacts in many fields, including computational cognitive modeling of decision making, although these applications have not yet been theoretically related to each other. This work introduces a categorization of applications of Generative Artificial Intelligence to cognitive models of decision making.

**Methods:**

This categorization is used to compare the existing literature and to provide insight into the design of an ablation study to evaluate our proposed model in three experimental paradigms. These experiments used for model comparison involve modeling human learning and decision making based on both visual information and natural language, in tasks that vary in realism and complexity. This comparison of applications takes as its basis Instance-Based Learning Theory, a theory of experiential decision making from which many models have emerged and been applied to a variety of domains and applications.

**Results:**

The best performing model from the ablation we performed used a generative model to both create memory representations as well as predict participant actions. The results of this comparison demonstrates the importance of generative models in both forming memories and predicting actions in decision-modeling research.

**Discussion:**

In this work, we present a model that integrates generative and cognitive models, using a variety of stimuli, applications, and training methods. These results can provide guidelines for cognitive modelers and decision making researchers interested in integrating Generative AI into their methods.

## 1 Introduction

Cognitive models of decision making aim to represent and replicate the cognitive mechanisms driving decisions in various contexts. The motivation for the design and structure of cognitive models is based on various methods; some models focus on the connection to biological processes of the brain, while others aim to emulate more human-like behavior without a biological connection. However, these motivations are not exhaustive or mutually exclusive. In fact, many approaches seek to reconcile these objectives and integrate the various methods. This paper proposes a framework to apply Generative Artificial Intelligence (GAI) research methods to cognitive modeling approaches and evaluates the efficacy of an integrated model to achieve the varied goals of decision modeling research.

Generative Models (GMs) are a category of AI approaches that generate data, often corresponding to the input data type, covering textual, visual, auditory, motor, or multi-modal data (Cao et al., 2023). GMs have shown remarkable advances, in various domains, in the effective generation and representation of complex data, unattainable with conventional methods (Bandi et al., 2023). The large space of research in GAI methods can be daunting for cognitive modelers interested in applying these techniques to their models for various reasons. The complexity and variety of these approaches are one of the motivations of this work, where we additionally seek to provide insights on the methods for applying GAI to cognitive models of decision making.

Although GMs have shown impressive success in various data modalities relevant to decision science research, there are significant concerns about their utilization (Bommasani et al., 2021). This is due in part to the potential of biases present in language processing and generating models such as Large Language Models (LLMs) (Bender et al., 2021). Various lines of research have suggested close connections to GMs and biological processes in some contexts, such as Variational Autoencoders (VAEs) (Higgins et al., 2021) and Generative Adversarial Networks (GANs) (Gershman, 2019). However, there is a general lack of understanding of how GMs integrate with decision making in a biologically plausible manner. Due to this lack of clarity on the relationship between GMs in decision making and biological realism, careful consideration must be given when choosing integrations with cognitive models aiming at reflecting biological realities.

Previously, the integration of GMs with cognitive models of decision making has been largely done on a case-by-case basis aimed at satisfying the needs of particular learning tasks (Bates and Jacobs, 2020; Malloy et al., 2022a; Xu et al., 2022), for a complete list of these approaches, see the Supplementary material. Consequently, there is an absence of a comprehensive framework for potential methods to integrate GMs and cognitive models of decision making. Understanding the impact of different integration methods is important, especially given the risks associated with improper application of AI technologies, particularly new ones within decision-making systems (Navigli et al., 2023) and the broader social sciences (Bommasani et al., 2021). Thus, elucidating these integration strategies has significant implications for ensuring the responsible and effective deployment of AI in decision-making contexts.

To address the challenges posed by GMs, one approach is to construct an integration of GMs and cognitive models in a way that allows for effective testing of component parts. This research introduces a novel application of GAI research and cognitive modeling of decision making, as well as a categorization of the different features of past integrations. This categorization not only aims at informing the design of future integrations, but also provides a means of comparison between different integration approaches. Based on this framework, we offer an ablation study to compare the integration of GMs into cognitive models. This method enables a thorough analysis of the individual components of these integrations, shedding light on how different integration methods affect behavior.

## 2 Related work

### 2.1 Cognitive architectures and instance-based learning theory

Several Cognitive Architectures (CAs) have been developed and applied to explain and predict reasoning, decision making, and learning in a variety of tasks, including SOAR (Laird et al., 1987), CLARION (Sun, 2006), and ACT-R (Anderson et al., 1997). Among these, ACT-R has been the basis for many other frameworks and theories that have emerged from the mechanisms it proposes. In particular, Instance-Based Learning Theory (IBLT) is based on an ACT-R mechanism that represents the process of symbolic cognition and emergent reasoning to make predictions from memory and determine human learning and decision making (Gonzalez et al., 2003).

Instance-Based Learning Theory (IBLT) is a cognitive approach that mirrors human decision-making processes by relying on the accumulation and retrieval of examples from memory instead of relying on abstract rules (Gonzalez et al., 2003). IBL models serve as tangible applications of IBLT tailored to specific tasks, encapsulating decision contexts, actions, and rewards pertinent to particular problem domains. These models learn iteratively from previous experiences, store instances of past decisions, and refine the results through feedback from the environment. Subsequently, IBL models leverage this repository of learned instances to navigate novel decision challenges. The adaptive nature of IBL models makes them particularly effective in contexts characterized by variability and uncertainty, as they can adapt flexibly to new situations by drawing parallels with past encounters. In particular, IBL models excel at capturing intricate patterns and relationships inherent in human behavior, a feat often challenging for explicit rule-based representations. Thus, IBLT stands as an intuitive framework to clarify how humans assimilate knowledge from experience and apply it to novel decision-making scenarios (Gonzalez, 2023).

In this research we selected IBLT due to its theoretical connection to the ACT-R cognitive architecture and its wide and general applicability to a multitude of tasks. IBL models have demonstrated fidelity to human decision making processes and have demonstrated their efficacy in various domains, including repeated binary choice tasks (Gonzalez and Dutt, 2011; Lejarraga et al., 2012), sequential decision-making (Bugbee and Gonzalez, 2022), theory of mind applications (Nguyen and Gonzalez, 2022), and practical applications such as identifying phishing emails (Cranford et al., 2019), cyber defense (Cranford et al., 2020), and cyber attack decision-making (Aggarwal et al., 2022).

IBL models make decisions by storing and retrieving instances *i* in memory M. Instances are stored for each decision made by selecting options *k*. Instances are composed of features *j* in the set F and utility outcomes *u*_*i*_. These options are observed in an order represented by the time step *t*, and the time steps in which an instance occurred is given *T*(*i*).

Each instance *i* that occurred at time *t* has an activation value, which represents the availability of that instance in memory (Anderson and Lebiere, 2014). The activation is a function of the frequency of occurrence of an instance, its memory decay, the similarity between instances in memory and the current instance, and noise. The general similarity of an instance is represented by summing the value *S*_*ij*_ over all attributes, which is the similarity of the attribute *j* of instance *i* to the current state. This gives the activation equation as:


(1) $$A_{i}(t)=\ln(\sum_{t^{\prime }\in T_{i}(t)}{\left(t-t^{\prime }\right)}^{-d})+\mu \sum_{j\in ℱ}\omega_{j}(S_{ij}-1)+\sigma \xi$$

The parameters that are set either by modelers or set to default values are the decay parameter *d*; the mismatch penalty μ; the attribute weight of each *j* feature ω_*j*_; and the noise parameter σ. The default values for these parameters are (*d* = 0.5, μ = 1, ω_*j*_ = 1, σ = 0.25), which are based on previous studies on dynamic decision making in humans (Gonzalez and Dutt, 2011; Lejarraga et al., 2012; Gonzalez, 2013; Nguyen et al., 2023).

The probability of retrieval represents the probability that a single instance in memory will be retrieved when estimating the value associated with an option. To calculate this probability of retrieval, IBL models apply a weighted soft-max function to the memory instance activation values *A*_*i*_(*t*) (Equation 1) giving the equation:


(2) $$\begin{array}{l} P_{i}(t)=\frac{\exp A_{i}(t)/\tau }{{\sum^{\text{​}}}_{i^{\prime }\in {ℳ}_{k}}\exp A_{i^{\prime }}(t)/\tau } \end{array}$$

The parameter that is either set by modelers or set to its default value is the temperature parameter τ, which controls the uniformity of the probability distribution defined by this soft-max equation. The default value for this parameter is $\tau =\sigma \sqrt{2}$.

The blended value of an option *k* is calculated at time step *t* according to the utility outcomes *u*_*i*_ weighted by the probability of retrieval of that instance *P*_*i*_ (Equation 2) and summing over all instances in memory $M_{k}$ to give the equation:


(3) $$\begin{array}{l} V_{k}\left(t\right)=\underset{i\in M_{k}}{\sum }P_{i}\left(t\right)u_{i} \end{array}$$

IBL models use this Equation (3) to predict the value of options in decision-making tasks. These option blended values are ultimately used to determine the behavior of the IBL model, by selecting from the options currently available the choice with the highest estimated utility. The specific notation for these IBL model equations are described in the python programming package PyIBL (Morrison and Gonzalez, 2024).

### 2.2 Generative Artificial Intelligence

Recent methods in Generative Artificial Intelligence (GAI) have shown impressive success in a variety of domains in the production of natural language (Brown et al., 2020), audio (Kim et al., 2018), motor commands (Ren and Ben-Tzvi, 2020), as well as combinations of these through multi-modal approaches (Achiam et al., 2023). This is done through the training of Generative Models (GMs) which take as input some stimuli, often of the same type as the output, and learn to generate text, audio, and motor commands based on the input and training method. In this work, we focus on the processing of visual and natural language information through the formation of representations achieved by GMs that are useful for cognitive modeling.

Visual GMs form representations of visual information and are originally structured or can be altered to additionally generate utility predictions that are useful for decision-making tasks (Higgins et al., 2017). These utility predictions generated by visual GMs have previously been applied to the prediction of human learning and decision making in contextual bandit tasks (Malloy et al., 2022a), as well as human transfer of learning (Malloy et al., 2023). Our approach is agnostic to the specific GM being used, which means that it can be applied to comparisons of different visual GMs to compare their performance.

#### 2.2.1 Representing data with GMs

The first of two desiderata to integrate GM in cognitive modeling of decision making was to relate models to biological processes in humans and animals. Here, this is understood within the context of representing data with GMs in a manner similar to that represented in biological systems. Recent research on GM-formed data representations has demonstrated close similarities to biological systems (Higgins et al., 2021), motivating their integration into cognitive models that are interested in similarity to biological cognitive systems.

An example of such a GM that is used in this work is the β-Variational Autoencoder (β-VAE) (Higgins et al., 2016, 2017) which learns representations that have been related to biological brain functioning, achieved by comparing the activity of individual neurons in the inferotemporal face patch of Macque monkeys to learned model representations when trained on images of human faces (Higgins et al., 2021). The format of these representations specifically is defined by a multi-variate Gaussian distribution that is sampled from to form a latent representation, which is fed through the decoder neural network layers to result in a lossy reconstruction of the original stimuli. The training of these models includes a variable information bottleneck controlled by the β parameter. This information-bottleneck motivation of these models has been associated with cognitive limitations that impact decision making in humans, resulting in suboptimal behavior (Bhui et al., 2021; Lai and Gershman, 2021).

These representations have been related to the processing of visual information from humans in learning tasks (Malloy and Sims, 2022), as they excel in retaining key details associated with stimulus generation factors (such as the shape of a ball or the age of a person's face) (Malloy et al., 2022b). Although we employ β-VAEs in this work, there are many alternative visual GMs that are capable of forming representations useful for decision making. This includes visual generation models including Generative Adversarial Networks (GANs) and Visual Transformer (ViT) based models. In our previous work, we performed a comparative analysis of various integrations with an IBL model (Malloy et al., 2023) and demonstrated that each can be effectively integrated with IBL to produce reasonable human-like behavior, but that information-constrained methods like the β-VAE are most accurate.

#### 2.2.2 Decision making with GMs

The second of two desiderata to integrate GMs into cognitive models of decision making is generating behavior that is similar to biological systems. This possibility is most salient in cases where GMs are capable of producing complex data, such as text, speech, or motor commands, which alternative models are not equipped to produce. However, in many cases making decisions in specific contexts with pre-trained GMs can be difficult due to the large size and training time of models such as BERT (Kenton and Toutanova, 2019), GPT (Radford et al., 2018), and PaLM (Chowdhery et al., 2023), as these models are not trained to explicitly make decisions.

Many recent approaches have applied GMs and their component structures (such as transformers Chen et al., 2021 or variational autoencoders Higgins et al., 2017), directly to decision making, in machine learning research. In Kirsch et al. (2023), the authors apply transformer models to learn generalizable behavior that can be applied in a variety of reinforcement learning (RL) domains, such as robotics (Brohan et al., 2023), grid-based environments (Li et al., 2022), and video games (Reid et al., 2022).

Other approaches apply feedback to RL models through the use of LLMs (McDonald et al., 2023; Wu et al., 2023), to provide a similar model learning experience as methods such as RL with human feedback (Griffith et al., 2013), without the need to collect human judgements. Offline RL has also been investigated through the integration of LLMs to reduce the need for potentially computationally expensive online learning (Shi et al., 2023). Beyond RL-based methods, some approaches draw some inspiration from cognitive architectures by using a similarity metric to a history of outputs to inform new choices such as the Generative Agents approach (Park et al., 2023).

### 2.3 Integrations of generative models and cognitive models in decision making

Previous research has explored numerous instances of integrating GMs and cognitive models, but these efforts have often been confined to single domains such as language, visual processing, or motor control. Additionally, the integration of GMs and cognitive models has typically been done for a single task or set of closely related tasks, mainly used to address a specific limitation within a cognitive model. These related applications span a diverse range of domains, including prediction of human transfer of learning (Malloy et al., 2023), phishing email detection (Xu et al., 2022), motor control (Taniguchi et al., 2022), auditory learning (Beguš, 2020), and multi-modal learning (Ivanovic et al., 2018).

Integrating GMs and cognitive models can be done in various ways: by replacing an existing functionality, enhancing a sub-module, or introducing a novel ability to the model. For example, LLMs have been proposed as potential knowledge repositories within cognitive models. These repositories can be accessed when relevant knowledge is required (Kirk et al., 2023), similar to a human-generated repository of general knowledge such as ConceptNet (Speer et al., 2017). In particular, ConceptNet has previously been integrated into a cognitive modeling framework for tasks such as answering questions (Huet et al., 2021).

Another recent approach used LLMs to produce highly human-like interactions between agents in a multi-player game involving natural language communication (Park et al., 2023). Although this model did not directly implement cognitive architectures, it did use inspiration from several architectures that were previously applied to multiplater games like Quakebot-SOAR (Laird, 2001) and ICARUS (Choi et al., 2007). This was done by incorporating a database of encodings of previously observed textual stimuli and then comparing them based on similarity (Park et al., 2023). Human-like language generation has also been investigated by applying GM techniques (Friston et al., 2020).

Outside the context of language models, some work has provided evidence for connections between human visual information processing and Generative Adversarial Networks (GANs) (Goetschalckx et al., 2021). Another method applied VAEs to modeling working memory formation in a task that required identifying the type of fault in a geological education task (Hedayati et al., 2022). In social science research, GMs have been applied on a range of tasks in replicating and reproducing well-studied phenomena in human social behavior (Aher et al., 2023; Ziems et al., 2023). In Hedayati et al. (2022), the authors employ a VAE to form representations used by a Binding Pool (BP) model (Swan and Wyble, 2014) to predict the categorization of visual stimuli.

#### 2.3.1 Categories of integrating generative models and cognitive models in decision making

Table 1 shows a selection of the most relevant previous approaches to the integration of GM and cognitive models of decision making and learning. A longer version of this analysis of previous methods is included in the Supplementary material, including some of the applications of GMs in decision science or machine learning that did not directly utilize cognitive modeling or did not predict human behavior.

**Table 1:** Comparison of previous applications of integrating GMs into cognitive models based on our proposed categorization.

	**Generative actions**	**Generative memories**	**Stimuli type**	**Cognitive model**	**GM type**	**GM training**
GINGER (proposed)	✔	✔	Textual or Visual	IBL	VAEs, LLMs	*Ad-hoc*, Pretrain
Mitsopoulos et al. (2023b)	✗	✔	Textual	ACT-R	LLMs	Pretrain
Malloy et al. (2023)	✗	✔	Visual	IBL	VAEs, GANs	*Ad-hoc*
Malloy et al. (2022a)	✔	✗	Visual	RL	VAEs	*Ad-hoc*
Xu et al. (2022)	✗	✔	Textual	IBL	LLMs	Pretrain
Hedayati et al. (2022)	✗	✔	Visual	BP	VAEs	*Ad-hoc*
Higgins et al. (2021)	✔	✗	Visual	RL	VAEs	*Ad-hoc*
Bates and Jacobs (2020)	✔	✗	Visual	None	VAEs	*Ad-hoc*

Additional comparisons to models that are not applied to biological cognition are enumerated in an extended table in the Supplementary material. Green check marks indicate that a model or method contains the related generative method. Red x marks indicate that a model or method does not contain the related generative method.

Previous approaches are categorized based on the following features: (1) Generative Actions: whether the GM is used to generate the actions executed by the agent; (2) Generative Memories: Whether the memory representations used by the cognitive model are generated by a GM; (3) Stimuli Type: the types of stimuli the GM is capable of processing; (4) Cognitive Model Type: the type of cognitive model that is used as a base for integration; (5) GM Type: the type of GM that is integrated into the cognitive model; and (6) GM Training: Whether the GM is pre-trained on a large existing corpus, as is done in foundation models, or trained in a tailored manner to solve a specific modeling task.

These features for evaluating existing models are motivated in part by *The Common Model of Cognition* (Laird et al., 2017), which describes the commonalities that cognitive architectures such as SOAR and ACT-R have in terms of their connections of different cognitive faculties. The common model of cognition reviews the history of cognitive model comparisons, based on their method of producing actions, memories, types of perception items, and how these faculties were connected.

Mitsopoulos et al. (2023b) propose an integration of GMs into their “psychologically valid agent” framework, which is rooted in ACT-R and IBLT. This framework has been instrumental in modeling and predicting COVID masking strategies, as demonstrated in their study on this topic (Mitsopoulos et al., 2023a). Another architecture, CogNGen (Ororbia and Kelly, 2023), incorporates MINERVA 2 (Hintzman, 1984) as a short-term memory module while performing other cognitive faculties using both predictive coding (Rao and Ballard, 1999) and neural generative coding (Ororbia and Kifer, 2022). The efficacy of this architecture has been demonstrated in various grid-world tasks (Chevalier-Boisvert et al., 2018), demonstrating improved success in challenging escape-room style environments.

Connecting cognitive models with GMs to produce memory representations of decision making tasks has been explored in Malloy et al. (2023), which compared Generative Adversarial Networks (GANs), Variational Autoencoders (VAEs) and Visual Transformers (ViTs), in their ability to integrate with an IBL model. This work was inspired by previous applications of GMs in modeling biological decision making, such as Higgins et al. (2021). Another approach which has incorporated LLMs with instance based learning was presented in Xu et al. (2022), which involved LLM model representations of phishing emails used to predict human decision making in an email categorization task.

## 3 Proposed model

### 3.1 Generation INformed by Generative Environment Representations (GINGER)

In this work, we propose a method that integrates GMs into both the action and memory generation of a cognitive agent based on IBLT. This integration of GMs and IBL models can process either textual or visual information which is achieved by leveraging Variational Auto-Encoders or Large language Models. The result is a method of Generation INformed by Generative Environment Representations (GINGER).

**Figure 1:**
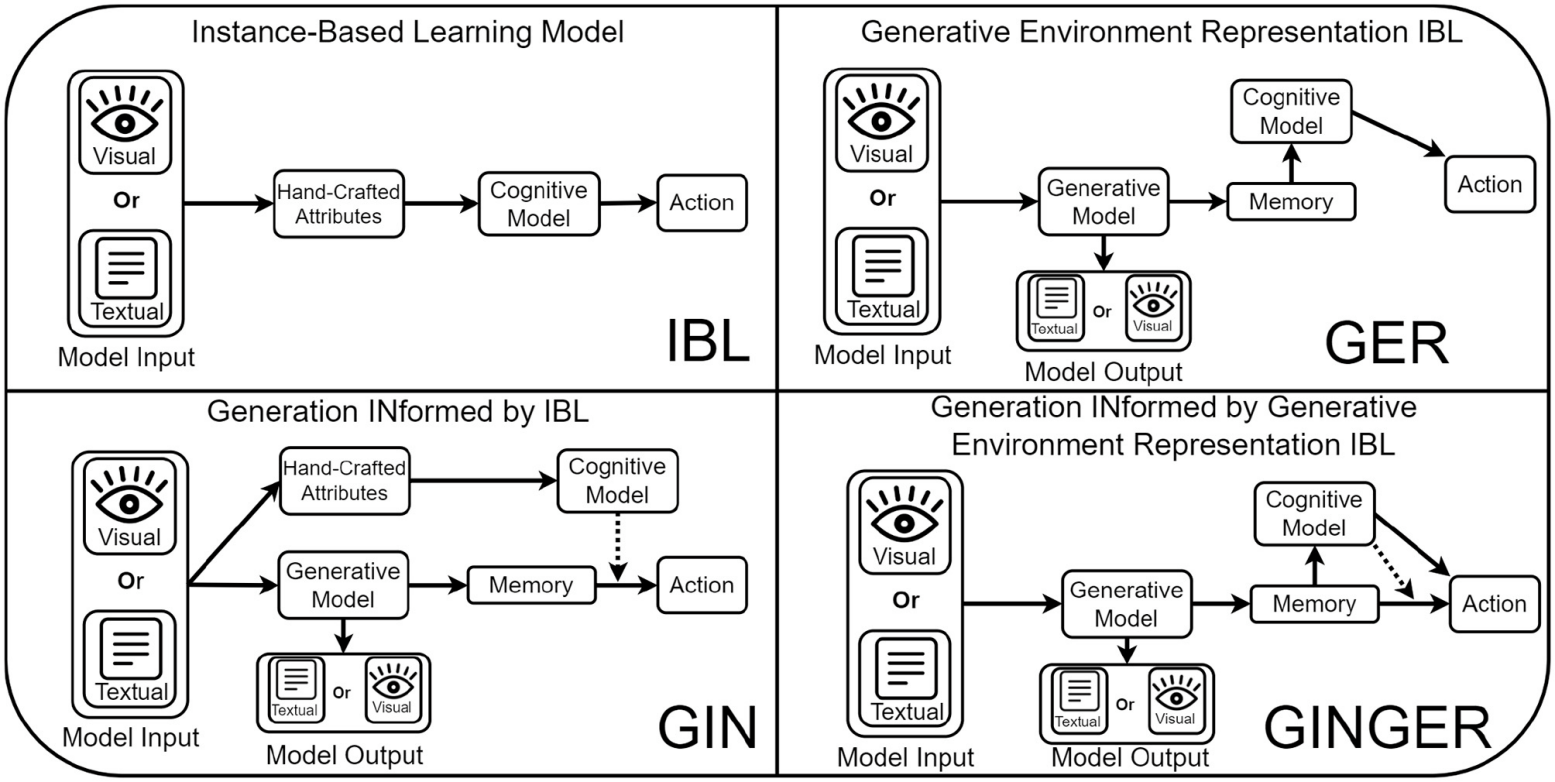
Comparison of our proposed GINGER model (**bottom right**) and an ablation of three alternative IBL based models. In the **top-left** is the basic IBL model which predicts decision making in visual or text based tasks in terms of hand-crafted attributes to produce actions. In the **bottom-left** is the Generation INformed by IBL model, which makes predictions of actions using a neural network that takes as input generative model representations and is trained according to an IBL model (dashed line). The **top-right** shows the Generative Environment Representation model, which makes predictions using an IBL model that uses features defined by the generative model. Finally, the full GINGER model combines these two approaches, predicting actions by evenly weighing GINIBL and GERIBL action predictions.

In Figure 1, we outline a general schematic of our proposed GINGER model. The first step of this process is for the GM model input to be processed by the model. In the experiments used for this work, this includes textual and visual information, but could be applied to others. From this input, the GM produces some model output and representations of the model input that is used as the memory of the GINGER model. This is used by the cognitive model, either as a part or as the whole of the state representation. From these two action prediction methods, the GINGER model produces two action outputs, which are resolved based on the specifics of the environment, such as averaging for utility prediction.

There are two optional connections between GMs and cognitive models that are not investigated in this work and are instead left for future research. The first is the connection from the model output to the action being performed. While the generation of utility predictions is always informed by cognitive model predictions (by training the actions based on cognitive model predictions), it is also possible to include the GM output (text, motor commands, etc.) as the whole or a part of the action performed. Secondly, the cognitive model and GM can optionally be connected from the cognitive model into the GM input, such as by using predictions from the cognitive model as a part of the GM input (e.g., as the prompt of a LLM) to inform how representations and outputs should be formed.

#### 3.1.1 Generative actions

The first part of the GINGER model name, “generation informed by” refers to the sharing of utility predictions made by the cognitive model when training the utility prediction of the generative model. Action generation is accomplished by directly generating utility predictions that are used in decision making tasks to determine the action with the highest utility based on a specific stimulus. This can be achieved in two different ways depending on whether the GM is a pre-trained foundation model or an ad-hoc trained model for a specific task.

In the case of ad-hoc trained models, the models themselves have been adjusted to generate utility predictions and are trained using the cognitive model. For instance, a β-Variational Autoencoder (β-VAEs) model which typically produces reconstructions of original stimuli can be adjusted to additionally predict utility, as was done in previous methods (Higgins et al., 2017; Bates and Jacobs, 2020; Lai and Gershman, 2021; Malloy et al., 2022a). Then, instead of training the model to predict actions based on reward observations from the environment, it is trained to match the predictions of the cognitive model. β-VAEs are trained to produce as accurate reconstructions as possible, given the size of the latent representation and its informational complexity, measured by KL-divergence, which is penalized through the β parameter. This means that adjusting the β parameter to individual cognitive abilities can result in more human-like predictions of actions based on model representations (Bates and Jacobs, 2019, 2020; Malloy et al., 2022b).

In the case of pre-trained or foundation models, the models cannot be easily adjusted after training prior to integration with cognitive models. For that reason, when integrating pre-trained LLMs or other foundation models, our GINGER approach uses representations learned by these models as input to a separate utility prediction neural network. The structure and precise training of these models is left to the discretion of cognitive modelers according to the demands of the learning task under investigation. In our work, we use a simple 2 layer fully connected network with 64 units to predict the utility associated with these representations. See the Supplementary material for more details on this training approach.

#### 3.1.2 Generative memories

The second part of the GINGER name, “generative environment representations”, refers to the creation of stimuli representations that are created based on the requirements of the learning task to capture the stimuli type of interest. This reliance on representations formed by GMs allows for either total reliance on representations, or adding the representation as an additional feature. When applying these representations to IBL, we determine the similarity *S*_*ij*_ in the calculation of the activation function (see Equation 1) through an integration of a similarity metric defined by the training of the GM Sim_GM_ as follows:


(4) $$\begin{array}{l} \begin{array}{c} A_{i}\left(t\right)=\ln\;(\underset{t^{\prime }\in T_{i}\left(t\right)}{\sum }{\left(t-t^{\prime }\right)}^{-d})+\mu \underset{j\in F}{\sum }\omega_{j}\left(S_{ij}-1\right)+\sigma \xi \\ S_{ij}={\text{Sim}}_{\text{GM}}\left(p\left(z_{i}|k_{i}\right),p\left(z|k\right)\right) \end{array} \end{array}$$

Formally, GMs process some input *x*, which can be visual, textual, auditory, or multi-modal input, and produce some output *y* based on that input. During this generation, these models form representations of the input *z* that can vary in structure, such as the multivariate Gaussian distributions used by β-VAEs or word vector embedding used by LLMs. In our model, we consider the option or part of the option relevant for modeling *k* to be the input to the GM. This allows for the formation of representations *z* based on these options. The similarity of options can be instead calculated based on these representations of current options *p*(*z*|*k*) and representations of options stored in the IBL model memory *p*(*z*_*i*_|*k*_*i*_). The similarity of these representations is defined by the training method of the GM, used as a metric of similarity (Sim_GM_).

In some GMs such as conversational LLMs, the output *y* is trained to match with subsequent textual tokens in a conversation or other language domain. In other types of GMs like Variational Autoencoders the models are trained such that the output *y* is as close to the input *x* as possible given the information constraint imposed by the model. These two types of models are used in our comparison of different methods of integrating GMs, but alternative GM structures and training methods can also be integrated with our proposed modeling approach.

The generation of internal representations is a requirement in a sense for GMs as they must form some representation *z* based on the input *x* in order to process it. As with the model output *y*, the structure of these internal representations *z* varies between different GMs. In the case of LLMs, these internal representations are structured as word vector embeddings. This allows for measures of similarity (Sim_GM_ in Equation 4) based on cosine similarity, which is conceptually similar to a high-dimensional distance metric. In the case of β-VAEs, these representations take the form of high-dimensional Gaussian distributions which are sampled from and fed through the subsequent layers of the model to form the reconstructed version of the original stimulus. With these types of representations, it is possible to measure similarity in terms of the KL-divergence of these representations.

In both cases, these GMs provide a meaningful representation of the model input, as well as a method of comparing these representations to other inputs. This is highly relevant for integration with an IBL method since the similarity of instances needs to be calculated to determine a memory activation, which is easily achieved through the use of the existing similarity metric required by the training of the GM itself. The next sections on generative action production and generative memory production will further detail how the representations formed by GMs are used in the IBL cognitive model, as well as how the IBL and GM are integrated in an interdependent manner that affords improvements to both models.

## 4 Model ablation

This work proposes a comparison of different methods of integrating GMs into cognitive models or architectures, through an ablation study comparing the categorizations described in Table 1. To do this, we use the Instance-Based Learning (IBL) model of dynamic decision making (Gonzalez et al., 2003). As opposed to a comparison of our proposed model against a highly similar model that instead is based on a different cognitive model or GM, or has a different method of integrating GMs and cognitive models, we are interested in providing insight to cognitive modelers interested in applying GMs to their own approaches, and as such adopt an ablation analysis of GINGER.

This ablation is based on the two key features of GINGER, the ‘Generative Environment Representations' which are related to the generation of cognitive model memory representations, and the ‘Generation Informed' by cognitive models, allowing for the actions selected by GMs to take information from cognitive models. Ablating away the generative environment representations results in a model that only uses generation informed by cognitive models (GIN). Ablating away the generation informed by cognitive models results in a model that only uses generative environment representations (GER). Finally, ablating both away results in the baseline Instance Based Learning (IBL) model which makes predictions using hand-crated features of tasks.

These four models (GIN, GER, GINGER, and IBL) form the baseline for our ablation comparisons in three experimental contexts involving different types of stimuli and complexities. The following sections detail these experiments as well as comparisons of the performance of the proposed model and the ablated versions. Participant data from these experiments and all trained models, modeling result data, and code to replicate figures is collected into a single OSF repository.^1^

### 4.1 Contextual bandit task

**Figure 2:**
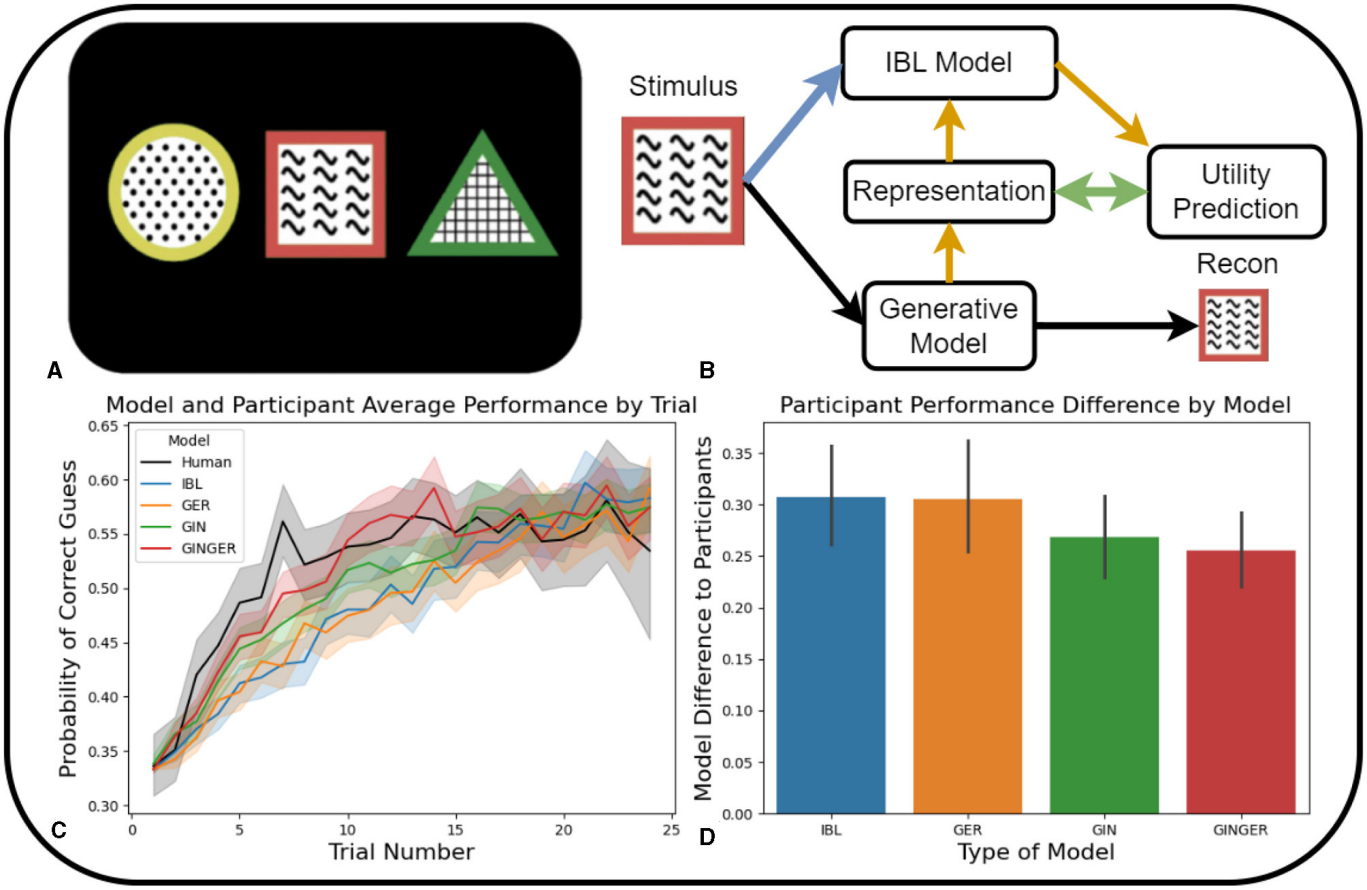
In **(B–D)**, Blue is the IBL model, Orange is the Generative Environment Representation IBL model, Green is the Generation Informed by IBL model and Red is the full Generation INformed by Generative Environment Representation IBL model. **(A)** Example of the stimuli shown to participants when making a decision on which of the features is associated with a higher probability of receiving a reward. **(B)** Schematic of the input of a single stimuli option into the generative and cognitive model making up the full GINGER model. The colored lines indicate the remaining connections of the ablated versions of the models. **(C)** Learning rate comparison of human participants and four ablated model versions in terms of probability of correctly guessing the option containing the feature of interest. **(D)** Average model difference to participant performance calculated by mean residual sum of squares for each participant. Error bars represent 95% confidence intervals.

The experiment was originally conducted at the Niv Neuroscience Lab at Princeton University (Niv et al., 2015). Participants were presented with three options, each distinguished by a unique combination of shape, color, and texture. Shapes included circular, square, and triangular forms; colors ranged from yellow, red, and green; and textures were dotted, wavy, and hatched (see Figure 2A). In every trial of the task, all of the 9 possible features appeared once within each option, ensuring that there will always be an option of each color, shape and texture. The features within the options were randomized to prevent repetitions in each position (left, middle, right). Participants had 1.5 seconds to make their selection, followed by a brief display (0.5 seconds) of the chosen option and the feedback showing the point reward (0 or 1). Then a blank screen was displayed for 4–7 seconds before the next stimulus.

During a single episode of the task, one of the nine features is selected as the feature of interest, and selecting the option with that feature increases the likelihood of receiving a reward. Episodes lasted approximately 20-25 trials before transitioning to a new feature of interest. The reward in this task is probabilistic, and selecting the feature of interest results in a 75% chance of receiving a reward of 1 and a 25% chance of receiving a reward of 0. When selecting one of the two options without the feature of interest resulted in a 25% chance of receiving a reward of 1 and a 75% chance of observing a reward of 0. Given the three possible options, the base probability of selecting the option with the feature of interest was 1/3.

#### 4.1.1 Cognitive modeling

The contextual bandit task serves as a benchmark to compare the three approaches to integrating GMs into cognitive decision-making models. This simple task is useful to ensure that all integrations of GMs in cognitive modeling accurately capture human learning in basic learning scenarios. In Figure 2B, we present a visual representation of the GINGER model, which uses visual stimuli associated with one of the three options as input. First, this stimulus is fed into the GM. In this task, a modified version of a β-Variational Autoencoder is used to further predict the utility associated with stimuli based on the internal representations generated by the GM.

For the baseline IBL model, choice features consisted of shape, color, and texture. For each type of feature, the similarity metric was defined as 1 for identical features and 0 for all other features. The GER model used the β-VAE model representation as an additional feature with a unique similarity metric. The similarity metric of this additional feature was the β-VAE model representation distribution KL-divergence. The GIN model used the baseline IBL model to predict utilities of stimuli options and trained the utility prediction network using these values. The full GINGER model combined these two approaches of the GIN and GER models in this task. All four ablation models used the same predefined parameters for noise, temperature, decay, and as mentioned previously.

#### 4.1.2 Methods

The experimental methodology is reproduced from the original paper; for additional details, see Niv et al. (2015). This study involved 34 participants (20 female, 14 male, 0 non-binary) recruited from Princeton University, all aged 18 or older. Data from 3 participants were incomplete and thus not analyzed, and another 6 participants were removed due to poor performance. Participants had a mean age of 20.9 years and were compensated at a rate of $20 per hour. This experiment was approved by the Princeton University Institutional Review Board. The experiment was not preregistered. Participant data is accessible on the Niv Lab website.^2^

To evaluate the performance of the 4 model ablation of our proposed GINGER model, we compare the probability of a correct guess on each trial within an episode. Figure 2C shows the comparison between participant and model performance regarding the probability of selecting the option containing the feature of interest across trials 1–25. This graphical representation facilitates the visual comparison of the learning of which feature is associated with a higher probability of observing a reward, and the average performance at the end of each episode.

In addition to the trial-by-trial comparison of model and participant performance depicted in Figure 2C, our aim is to compare the overall similarity between them. This is done by measuring the difference in model performance with individual participant performance using the mean residual sum of squares *RSS*/*n* where n is the number of participants and $RSS=\overset{n}{\underset{i=1}{\sum }}{\left(y_{i}-p\left(x_{i}\right)\right)}^{2}$. This difference is calculated for each participant and trial within an episode and across all episodes in the experiment. These values are correlated with the Bayesian Information Criterion (BIC) calculated in terms of the residual sum of squares (RSS) as *BIC* = *n*ln(*RSS*/*n*)+*kln*(*n*) since all four models have 0 fit parameters (all are default values). The resulting values are averaged across all participants and presented in Figure 2D. Error bars in Figure 2D denote the 95% confidence intervals of the model difference from participant performance across each participant and trial of the task.

#### 4.1.3 Results

The initial comparison of model learning to participant behavior focuses on the probability of correct guesses as the trial number within increases, as shown in Figure 2C. Comparing the speed of learning to participants reveals that models that include the generative action selection (GIN and GINGER) demonstrate the fastest learning. Compared to the two versions of the GINGER model (IBL and GER) that do not make direct predictions of utility based on GM representations exhibit slower learning rates. This shows that in learning tasks that require fast updating of predicted utilities, directly predicting these values from GM representations and selecting actions accordingly results in more human-like learning progress.

The second set of results illustrated in Figure 2D, compares the average difference in model performance to participants performance. Among the four models compared, the GINGER model has the lowest deviation from participant performance and a performance difference similar to the GIN ablation model, which relies on predictions of utility derived from GM representations. The IBL and GER models, which make predictions based on hand-crafted stimuli features (IBL) and GM representations (GER), show the highest difference to participant performance. The unique feature of the GINGER model involves predictions of utility partially influenced by the GM-formed stimulus representation related to the IBL model's use of the features. However, by directly predicting utility based on representations, both the GINGER and GIN models are able to quickly update utility predictions.

In summary, the modeling results demonstrate that each approach to incorporating GMs in predicting human learning is viable, as none of the models performs worse than the IBL model, which does not use a GM. However, models that perform actions selected by the GM exhibit more human-like learning trends (Figure 2C) and a closer similarity to human learning (Figure 2D). While leveraging GM representations aims to improve generalization, the simplicity of this task imposes minimal demands on generalization, meaning that the speed of learning is more relevant in producing human-like learning. The next experiment paradigms will introduce an explicit generalization requirement for participants. This will enable a comparison of ablated models in a task where generalization performance is more important.

### 4.2 Transfer of learning task

This decision-making task involves learning the values associated with abstract visual stimuli and transferring that knowledge to more visually complex stimuli. Previous research comparing the IBL and the GER model demonstrated improved performance in transfer of learning tasks by introducing generative representations to the IBL model (Malloy et al., 2023). The higher performance of the GER model and its closer resemblance to human performance compared to the standard IBL model, raises questions about how our proposed GIN and GINGER models compare in replicating human-like behavior in this transfer of learning task.

**Figure 3:**
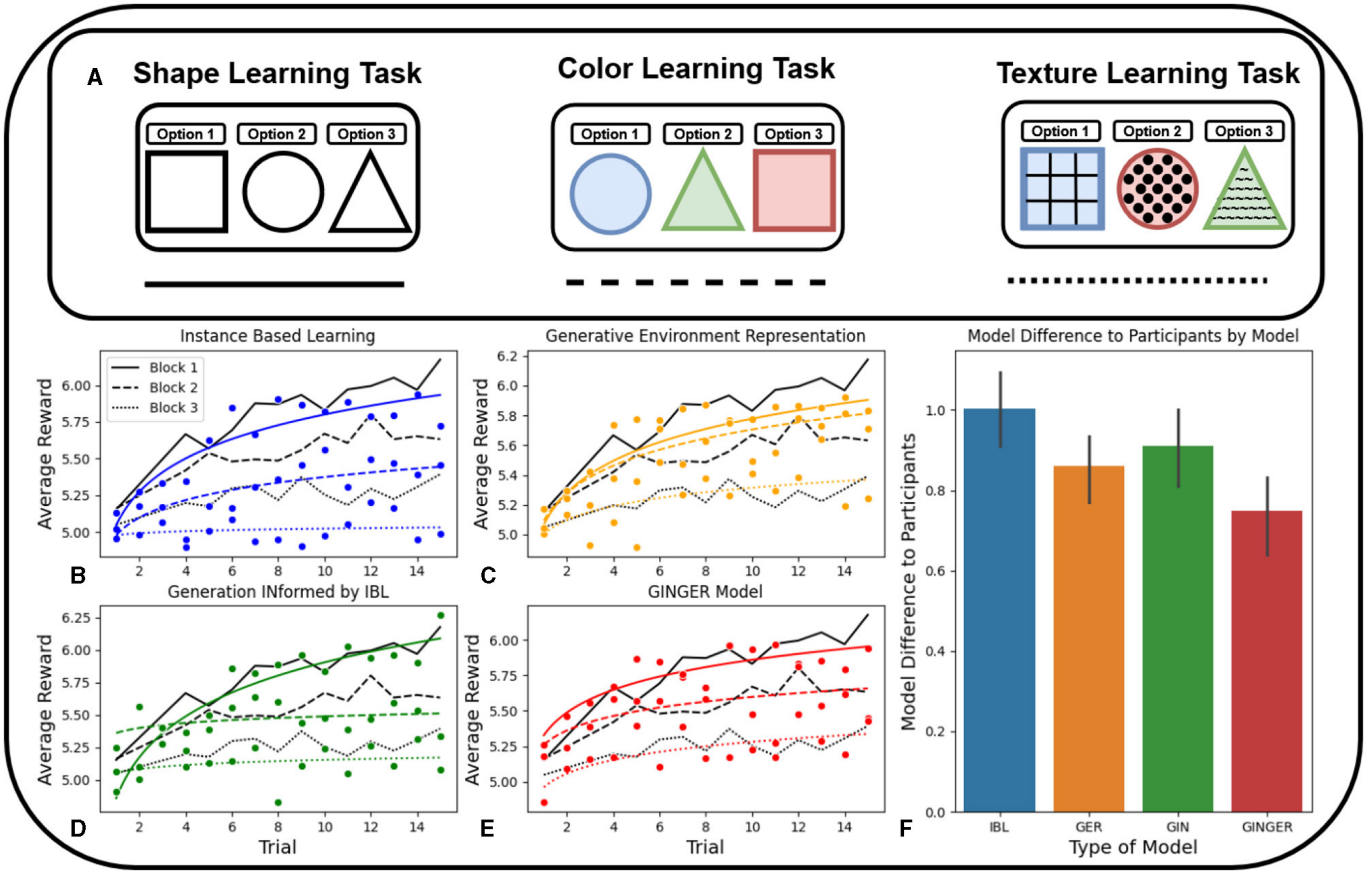
In **(B–F)**, Blue is the IBL model, Orange is the Generative Environment Representation IBL model, Green is the Generation Informed by IBL model, and Red is the full Generation INformed by Generative Environment Representation IBL model. **(A)** Example stimuli of one block of 15 trials for the shape, color, and texture learning tasks, adding to a total of 45 trials. **(B)** Performance of the model against human participants on each of the three learning tasks shown in **(A)**. **(C)** Comparison of GINIBL and human learning across the three learning tasks. **(D)** Comparison of GINGER and human learning across the three tasks. **(E)** Comparison of GERIBL and human learning across the three learning tasks. **(F)** Comparison of accuracy between model predicted learning and human performance calculated by mean residual sum of squares. Error bars represent 95% confidence intervals.

In this task, generalization performance is more relevant than learning speed in evaluating participants and cognitive models. This is due to the increase in task complexity over time. Initially, participants engaged in a contextual bandit task focused only on the shape feature (Figure 3A Left). After 15 trials the task complexity increases with the introduction of the color feature (Figure 3A Middle). Transitioning to the color learning task requires participants' ability to transfer knowledge from the shape learning task to determine the optimal option. This demands generalization from past experience to make future decisions in a related but not totally equivalent context. After these 15 trials of the color learning task, participants are introduced to the texture learning task (Figure 3A Right) which is similar to the structure of the first learning experiment (Niv et al., 2015).

#### 4.2.1 Cognitive modeling

The design of IBL baseline model features was identical to the first experiment, including the use of the shape, color, and texture features, baseline parameter values, and binary similarity metrics. One difference between this task and the previous one is that the GIN and GINGER utility prediction modules are only being trained using one portion of the data set at a time, first shape, then shape-color, then shape-color-texture. This means that predicting utility associated with a representation requires a high degree of generalization to adequately transfer from one task to the other.

#### 4.2.2 Methods

160 participants (86 female, 69 male, 2 non-binary) were recruited online through the Amazon Mechanical Turk (AMT) platform. All participants were over the age of 18 and citizens of the United States of America. Participants had a mean age of 40.5 with a standard deviation of 11.3 years. Participants were required to have completed at least 100 Human Intelligence Tasks (HITs) on AMT with at least a 95% approval on completed HITs. Six of the 160 recruited participants failed to submit data or failed to complete the task within a 1 hour limit, and were excluded from analysis. All results and analysis are done using the remaining 154 participants.

Participants received a base payment of $4 with the potential to receive a bonus of up to $3 depending on their performance in the task. The mean time to complete the task was 16.9 minutes, with a standard deviation of 5.8 minutes. This experiment was approved by the Carnegie Mellon University Internal Review Board. The experiment protocol was preregistered on OSF. Experiment preregistration, participant data, analysis, model code, and a complete experiment protocol are available on OSF.^3^ For a more complete description of experiment methods, see Malloy et al. (2023).

Participant's performance in this task can be measured in their ability to transfer knowledge from one learning task to the subsequent learning tasks. Three commonly used metrics for performance in transferring learned knowledge to subsequent tasks are jumpstart, asymptotic, and episodic performance (Taylor and Stone, 2009). Jump-start performance is defined as the initial performance of an agent on a target task. In the contextual bandit experiment used in this work, the jumpstart performance is calculated as the average of the first third observed utility in trials after the task switches. Asymptotic performance is defined as the final learned performance of an agent in a target task. In the transfer of learning experiment, the asymptotic performance is calculated as the average of the final three reward observations of participants. Episodic Performance is defined as the average performance over an episode; this measure is analogous to the total reward metric commonly used. This value is calculated as the average of the observed utility. These measures are used to compare model difference to participant behavior, and averaged over to produce the results shown in Figure 3F.

#### 4.2.3 Results

To assess transfer of learning for the three measures, we averaged the similarities between human and model performance in jumpstart, episodic, and asymptotic performance in the three learning tasks. This aggregation yields a single metric, providing a holistic evaluation of the fit between the model and human transfer of learning performance. This similarity is based on average residual sum of squares *RSS*/*n* calculations for each of the three measures of transfer of learning measures. This integrated measure of congruence is shown in Figure 3F, to facilitate a comparison across the four models. Importantly, these accuracy metrics are computed for each participant individually, ensuring the understanding of performance across the sample. Additionally, the same connection between average residual sum of squares and BIC can be made as in the first experiment, since again there are no fit parameters.

As in the contextual bandit task in Experiment 1, we first compare the four models by their speed of learning, and the similarity to human performance, shown in the four plots (Figures 3B–E). This is done for each of the three learning tasks that increase in complexity as the experiment progresses. This comparison shows that the GER and GINGER models have learning trend more similar to humans in the color and texture tasks compared to the IBL and GIN models. This is likely because of the fact that the representations of visual information used by the GER and GINGER models as features of the IBL model allow for improved generalization, which is a key feature of improving transfer of learning ability.

Comparatively, the IBL and GIN models show more human-like learning on the simple shape learning task before the transfer of learning ability becomes relevant. This mirrors the human-like learning achieved by these two models in the first experiment, but because the majority of this task relies more on generalization capability rather than the speed of learning, the end result is that the GER and GINGER models are better fits to human learning averaged across the entire experiment.

The next comparison of model performance is shown in Figure 3F which captures an aggregate average of the three transfer of learning metrics previously discussed. Overall, the IBL model is far more distant from human performance than the three ablation models. The GER and GIN models are about equally distant from human performance, as the GER model has relatively higher performance on the two transfer tasks while GIN model had better performance on the first task. The GINGER model, which combines the more human-like behavior on the first task observed by the GIN model, and the two transfer tasks by the GER model, produces the most human-like learning on average.

### 4.3 Phishing identification task

Phishing messages are emails that contain attempts to obtain credentials, transmit malware, gain access to internal systems, or cause financial harm (Hong, 2012). An important aspect of preventing these phishing emails from negatively impacting individuals and companies is through training programs to help people identify phishing emails more successfully (Singh et al., 2020). Cognitive models have been applied to predict and improve email phishing training (Singh et al., 2019; Cranford et al., 2021). The phishing email identification task is used to compare the ablation of our proposed model in how relevant each of its attributes is in conditions that include complex natural language stimuli.

**Figure 4:**
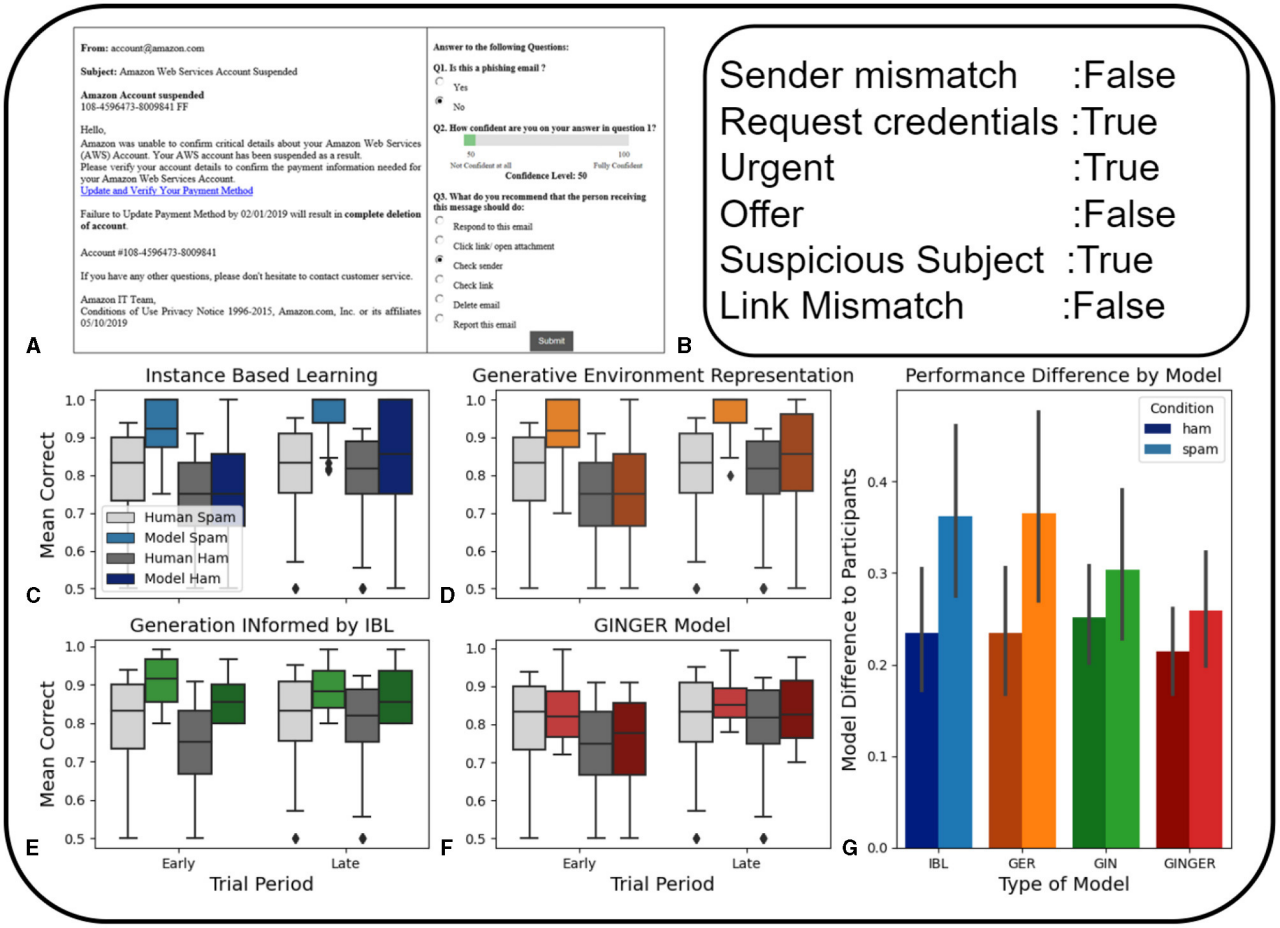
**(A)** Example email shown to participants and multiple choice and confidence selection from Singh et al. (2023). **(B)** Example of expert human coding of email features. In **(C–G)**, blue is the IBL model, orange is the GER model, green is the GIN model, and red is the GINGER model. Darker shading represents phishing emails, and lighter shading represents non-phishing ham emails. The four graphs on the bottom left show the performance of the models compared to human participants in correctly predicting ham and phishing emails. The error bars represent 95% confidence intervals. **(C)** IBL model performance compared to human participants. **(D)** Performance of the GER model compared to human participants **(E)** GIN model performance compared to human participants. **(F)** GINGER model performance compared to human participants. **(G)** Average difference in human participant performance on both ham and phishing email identification across each of the four models. This is calculated by the mean residual sum of squares. Error bars represent 95% confidence intervals.

We use a data set of human judgments on the phishing identification task (Figure 4A) that was originally collected in Singh et al. (2023) and is publicly available. The phishing identification task involved the presentation of phishing or safe emails. Participants indicated their guess as to whether the emails were safe or dangerous, their confidence rating, as well as a recommendation of an action to take when receiving this email, such as checking the link, responding to the email, opening an attachment, etc. (Singh et al., 2023). These details are described more fully in the section on experimentation methods.

#### 4.3.1 Cognitive modeling

The baseline IBL model for this task used binary hand-crafted features coded by human experts (Figure 4B) including mismatched sender, requesting credentials, urgent language, making an offer, suspicious subject, and a link mismatch. The other main difference in cognitive modeling of this experiment with the previous two is that a LLM model is used to form the representations used both as a feature of the task and directly trained to predict utilities.

These representations are embeddings of textual inputs formed by the OpenAI GPT based model “text-embedding-ada-002”. At the time of writing, this was the only text embedding model available on the OpenAI Application Programming Interface. This model generates representations of text inputs in the form of a vector of 1536 floating point numbers. The IBL similarity metric for these representations is calculated with the sklearn python package cosine similarity function, a commonly used metric when comparing sentence embeddings from large language models (Li et al., 2020).

Due to the high baseline performance of humans in this task, as a result of their experience in reading emails and their experience with phishing warnings, we use a random sampling of 10% of emails to pre-train all models under comparison. This allows for a more realistic comparison of the performance of these models in reflecting human decision making in this type of task.

#### 4.3.2 Methods

The experimental methods for this analysis are detailed in full in Singh et al. (2023). 228 participants were recruited online through the Amazon Mechanical Turk (AMT) platform. Participants were required to have completed at least 100 Human Intelligence Tasks (HITs) on AMT with at least a 90% approval rate. All participants were over the age of 18. Participants have a mean age of 36.8 with a standard deviation of 11.5 years. Four of the 228 participants failed attention checks and were excluded from the analysis. Participants were paid a base rate of $6 with the potential to receive a bonus of up to $3 depending on their performance. The mean time to complete this experiment was 35 minutes.

Experiment data were made available on request from the original authors and obtained by us after request. This experiment data included participant judgments in the task as well as the 239 emails that were classified by the researchers based features that were relevant to determine if the emails were phishing, referred to as spam, or non-phishing, referred to as ham. These features included whether the sender of the email matched the claimed sender; whether or not the email made a request of credentials; whether or not the subject line was suspicious; whether an offer was made in the email body; whether the tone of the email used urgent language; and finally whether a link in the email matched the text of the link. Textual data and email features are available on OSF^4^ and participant data are contained in our previously mentioned combined repository (see text footnote ^1^).

Participants' performance in this task can be measured in their ability to correctly identify phishing emails as phishing, and ham emails as ham. Splitting this classification by the type of email shown to participants allows for a comparison between the different amounts of phishing and ham emails that were shown to participants during the experimental conditions. Ideally, an accurate model of human learning in this task would be similar to human data for each of these types of categorization.

Accurately reflecting differences in experience with the identification of phishing emails from participants can be a difficult task for cognitive models. In IBL models, this could be done by using a set of different models with varied initial experiences with phishing and ham emails, which would result in differences in accuracy for categorizing these two types of email. However, to highlight the differences in ablation analysis, we do not differentiate the experience of models individually to better fit human performance, and instead use the same base-level experience across all models under comparison.

#### 4.3.3 Results

In this experiment, each of the four ablation models predicted the same emails shown to participants, in the same order. The ablation models used the values of the baseline parameters for all the parameters of the IBL model. Therefore, the total number of model runs was equal to the number of participants for each type of model ablation. Models were trained using a reward function of 1 point for correct categorization and 0 points for incorrect categorization. For the GIN and GER models, the utility prediction based on representations was done using the representation input of size 1536 followed by two layers of size 128 and finally an output of size 1. More details of this are included in the Supplementary material.

The performance of the GIN model is unique in that it predicts similarly high performance in the early and later trial periods for both types of emails (Figure 4E). This direct utility prediction based on representations can approach high accuracy from only a few examples. This is true for both phishing and ham emails, while humans display lower accuracy overall, and a large difference between accuracy in these two types of emails. It would be possible to reduce this training for the GIN model alone, however, this would mean that the GIN model is using less experience than the other models.

In general, taking an approach to fitting the training time of generative actions to human performance can be difficult for large representations sizes, as it requires multiple training periods that are computationally expensive. This is demonstrated by the difference in similarity with the results of human learning demonstrated by the GIN model. This is a key difference between the phishing email identification task, where the representation size is 1536, compared to the earlier tasks that used β-VAE model representations of size 9. However, these representation sizes are not considered to be a variable or fit parameter in any of the models. Thus, the same connection between the average residual sum of squares and BIC can be made as in the first experiment, since again there are no fit parameters.

The GINGER model has the highest accuracy to human performance (Figures 4C–G), as a result of it making predictions using both the GM and the email representations that are fed into an IBL model. This demonstrates the benefits of combining generative actions and generative memory formation, for tasks with complex natural language stimuli. This is especially true for tasks like this one where participants are likely to have previous experience from which they are drawing, as opposed to the two previous abstract tasks. This is because optimizing the GIN model alone to fit human participant performance is computationally expensive and the IBL and GER models are not able to learn the task quickly enough.

## 5 Discussion

This research proposes a model that demonstrates the benefits of integrating GMs and cognitive modeling and their potential applications. These techniques open new avenues in the investigation of human learning that were previously inaccessible to cognitive modelers. GAI has had a significant impact across many fields of study, motivating its application in cognitive modeling, especially in decision-making processes. However, before integrating GMs into cognitive models to represent and predict human decision making, it is important to investigate the relative impact that different methods of integration have on different tasks.

The GINGER model proposed in this work demonstrates the integration of GMs with cognitive models of decision making, such as IBL. Our approach demonstrates the accurate prediction of human learning and decision making across three distinct experimental paradigms, directly compared to real human decisions. These experiments encompass a diverse range of stimuli, spanning visual cues and natural language that varied in complexity, from learning abstract rewards to detecting phishing attempts in emails. The application of our GINGER model across these domains resulted in an improvement over traditional cognitive modeling techniques, clearly demonstrating the potential benefits of incorporating GMs into cognitive modeling frameworks.

In addition to our GINGER model, we developed a categorization approach that can be used to compare and relate different approaches to integrating GMs into cognitive modeling of decision making. Before current research, there were many applications of GMs in cognitive modeling, although typically this was done in a case-by-case manner to allow for use in a specific learning domain. Here, we compare the integration of GMs in cognitive modeling in six dimensions, including action generation, memory generation, stimuli, cognitive model type, generative model type, and training method.

This categorization motivated an ablation study to compare our proposed model with alternative versions that contained generative actions and memory and did not contain them. Additionally, the three experiment paradigms were chosen to further test the remaining categories of our analysis, to investigate the varied stimuli types, GM types, and training methods. The result is a comparison of model performance that spans many degrees of our proposed categorization. The first experimental comparison demonstrated faster and more human-like learning from models that produced decision predictions directly by GMs (GIN and GINGER). However, this faster learning was observed in a relatively simple task, raising the question of the potential benefits of GM memory formation (GER and GINGER) in more complex environments.

The second comparison of models through experimentation extended the analysis in the first experiment by introducing a generalization task that required transfer of learning. This is a useful comparison for our proposed model, as one of the often cited benefits of applying GMs to cognitive models is improved generalization. This raised the question of which method of integrating GMs would be more relevant for improving performance and the similarity to human participants in this task. The high generalizability of models that utilized GM memory representations confirmed this expectation, demonstrating the ability of cognitive models that integrate GM representations in reflecting human-like generalization.

In the third and final experimentation, we investigated the potential differences of our proposed modeling method when handling complex natural language in a phishing identification task. Comparing the performance of models with that of human participants in this task demonstrated a large difference between categorization accuracy for phishing and ham emails, which was difficult for the models to replicate. Previously, only cognitive models that used GM representations of textual information, such as phishing emails, have been used to predict human-like learning, but these results demonstrate that a combination of directly predicting values and GM representations is best for this type of task.

Overall, these results from the model comparison provide insight into the design of integration of generative modeling methods with cognitive models. Each of our experiments investigated a different area of human learning and decision making modeling and made important conclusions about how best to integrate GMs. Although the applications of our model comparison are broad, they do not represent every possible application of GMs to cognitive modeling. As demonstrated by our categorization, there are remaining stimuli types, generative models, and cognitive models that could be compared. One potential future area of research would be the application of multi-modal models and a comparison of learning with humans engaging in a multi-modal decision task.

While GMs have demonstrated a high degree of usefulness in cognitive modeling, the impact that they have on society at large has been called into question, as noted previously. One potential issue with the use of a model similar to one of the ones we used in the experiment on predicting how participants respond to phishing emails is that it could be used to improve the quality of phishing email campaigns. This is exacerbated by the potential to use GMs themselves to generate phishing emails. One potential future area of research is investigating how we can best mitigate these potential GM missuses. This could be done by tailoring phishing email education to the individual through the application of a model similar to the one we propose, which can allow students to experience phishing emails generated by GMs and learn from them.

## Data availability statement

Publicly available datasets were analyzed in this study. This data can be found here: https://osf.io/m6qc4/.

## Ethics statement

The studies involving humans were approved by Carnegie Mellon University Institutional Review Board. The studies were conducted in accordance with the local legislation and institutional requirements. The participants provided their written informed consent to participate in this study.

## Author contributions

TM: Formal analysis, Software, Visualization, Writing – original draft, Writing – review & editing. CG: Conceptualization, Funding acquisition, Methodology, Resources, Supervision, Writing – original draft, Writing – review & editing.

## Footnotes

^1^ https://osf.io/m6qc4/

^2^ https://nivlab.princeton.edu/data

^3^ https://osf.io/mt4ws/

^4^ https://osf.io/sp7d6/
