# Supplementary material for: Applying Generative Artificial Intelligence to cognitive models of decision making
Source: Front Psychol. 2024 May 3;15:1387948. doi: 10.3389/fpsyg.2024.1387948 (PMC11100990; doi:10.3389/fpsyg.2024.1387948)
Supplement: Supplementary file 1 [file Data_Sheet_1.PDF]

Supplementary Material

1 SUPPLEMENTARY MATERIALS

1.1 Generative Memories

1.1.1 Uβ-VAE Model Parameters

The UB-VAEs neural network model used for experiments was pretrained on the full stimuli space with 1000 epochs with a batch size of 100, and a mean utility for utility training. The encoder consisted of 4 convolutional layers, each with 32 channels, 4x4 kernels, and a stride of 2. This was followed by 2 fully connected layers, each of 256 units. The utility output layers consisted of two fully connected layers each of 64 units. The image input size was (3, 64, 64). The latent distribution consisted of one fully connected layer of 9 units parametrizing the mean and log standard deviation of 18 Gaussian random variables (or 1 unit in the first simulation example). The decoder architecture was simply the transpose of the encoder, but with the output parametrizing Bernoulli distributions over the pixels. ReLU activations were used throughout. An Adam optimizer was used for training with a learning rate of 5e-4.

1.1.2 LLM Model Representations

Representations used in the GERIBL model for the phishing identification task are created via the OpenAI Python Application Programming Interface using the text-embedding-ada-002 model. This generates representations of phishing and ham emails of size 1536. The IBL similarity metric of these representations is calculated with the sklearn python package cosine similarity function.

1.2 Generative Actions

1.2.1 Uβ-VAE Action Generation

Predictions of utilities associated from β-VAEs directly were done by modifying the training and structure of the β-VAE described above, to include 2 fully connected layers each of 64 units and a single utility prediction output. The input to the first of these fully connect layers a sample of size 9 from the internal representation layer of the model. Since this utility prediction was connected to the reconstruction layers, the accuracy of utility predictions could update the way that prior layers represented visual information.

1.2.2 LLM Action Generation

Predictions of actions from LLMs directly were determined by utility predictions in the GIN and GINGER models were predicted with a neural network of input size 1536 followed by 2 fully connected layers each of 64 units. In this task, 10% of emails, split evenly between ham and phishing, were used to train utility predictions, using the same set of emails for training on the GM memory formation previously described. ReLU activations were used throughout. A mean squared error optimizer was used for training with a learning rate of 1e-2. Since these LLMs were not updated during action prediction, the accuracy of utility predictions could not impact the way that prior layers represented visual information.

1.3 Extended Model Comparison

In this extended model comparison we include approaches in making decisions outside of the context of cognitive modeling of human decision making with human data. For this reason we have changed one of the comparison dimensions from cognitive model to decision model. The * Next to the (Higgins et al., 2021) model represents the use of behavioral data from Macaque monkeys instead of humans. The * next to the (McDonald et al., 2023) model represents the use of a reward function determined by the LLM that is applied to the RL model instead of direct generative actions.

Citation	Human Data	GM Action	GM Memory	Stimuli Type	Decision Model	GM Type	GM Training
GINGER (proposed)	✔	✔	✔	Textual or Visual	IBL	VAEs, LLMs	Ad-hoc, Pretrain
(Ororbia and Kelly, 2023)	✗	✔	✔	Motor	CogNGen	Tranformer	Ad-hoc
(Park et al., 2023)	✗	✔	✔	Textual	None	LLMs	Pretrain
(Oscar J. Romero, 2023)	✗	✔	✔	Textual	General	LLMs	Pretrain
(Zhou et al., 2023)	✗	✔	✗	Motor	RL	Diffusion	Ad-hoc
(McDonald et al., 2023)	✗	✔*	✗	Motor	RL	LLM	Pretrain
(Malloy et al., 2023)	✔	✗	✔	Visual	IBL	VAEs, GANs	Ad-hoc
(Oltramari, 2023)	✗	✗	✔	Textual	ACT-R	LLM	Pretrain
(Kirk et al., 2023)	✗	✗	✔	Motor	General	LLM	Pretrain
(Laird et al., 2023)	✗	✗	✔	Textual	General	LLM	Pretrain
(Mitsopoulos et al., 2023)	✔	✗	✔	Textual	ACT-R	LLMs	Pretrain
Hedayati et al. (2022)	✔	✗	✔	Visual	BP	VAEs	Ad-hoc
(Malloy et al., 2022)	✔	✔	✗	Visual	RL	VAEs	Ad-hoc
(Xu et al., 2022)	✔	✗	✔	Textual	IBL	LLMs	Pretrain
(Ajay et al., 2022)	✗	✔	✗	Motor	RL	Diffusion	Ad-hoc
(Hedayati et al., 2022)	✗	✔	✗	Visual	None	VAEs	Ad-hoc
(Higgins et al., 2021)	✔*	✔	✗	Visual	RL	VAEs	Ad-hoc
(Bates and Jacobs, 2020)	✔	✔	✗	Visual	None	VAEs	Ad-hoc

REFERENCES

Ajay, A., Du, Y., Gupta, A., Tenenbaum, J., Jaakkola, T., and Agrawal, P. (2022). Is conditional generative modeling all you need for decision-making? *arXiv preprint arXiv:2211.15657*

Bates, C. J. and Jacobs, R. A. (2020). Efficient data compression in perception and perceptual memory. *Psychological review* 127, 891

Hedayati, S., O'Donnell, R. E., and Wyble, B. (2022). A model of working memory for latent representations. *Nature Human Behaviour* 6, 709–719

Higgins, I., Chang, L., Langston, V., Hassabis, D., Summerfield, C., Tsao, D., et al. (2021). Unsupervised deep learning identifies semantic disentanglement in single inferotemporal face patch neurons. *Nature communications* 12, 6456

Kirk, J. R., Wray, R. E., and Laird, J. E. (2023). Exploiting language models as a source of knowledge for cognitive agents. *arXiv preprint arXiv:2310.06846*

Laird, J. E., Wray, R. E., Jones, S., Kirk, J. R., and Lindes, P. (2023). Proposal for cognitive architecture and transformer integration: Online learning from agent experience. In *Proceedings of the AAAI Symposium Series*. vol. 2, 302–306

Malloy, T., Du, Y., Fang, F., and Gonzalez, C. (2023). Generative environment-representation instance-based learning: A cognitive model. In *Proceedings of the 2023 AAAI Fall Symposium on Integrating Cognitive Architectures and Generative Models* (AAAI Press)

Malloy, T., Klinger, T., and Sims, C. R. (2022). Modeling human reinforcement learning with disentangled visual representations. *Reinforcement Learning and Decision Making (RLDM)*

McDonald, C., Malloy, T., Nguyen, T. N., and Gonzalez, C. (2023). Exploring the path from instructions to rewards with large language models in instance-based learning. In *Proceedings of the 2023 AAAI Fall Symposium on Integrating Cognitive Architectures and Generative Models* (AAAI Press)

Mitsopoulos, K., Bose, R., Mather, B., Bhatia, A., Gluck, K., Dorr, B., et al. (2023). Psychologically-valid generative agents: A novel approach to agent-based modeling in social sciences. In *Proceedings of the 2023 AAAI Fall Symposium on Integrating Cognitive Architectures and Generative Models* (AAAI Press)

Oltramari, A. (2023). Enabling high-level machine reasoning with cognitive neuro-symbolic systems. In *Proceedings of the 2023 AAAI Fall Symposium on Integrating Cognitive Architectures and Generative Models* (AAAI Press)

Ororbia, A. and Kelly, M. A. (2023). A neuro-mimetic realization of the common model of cognition via hebbian learning and free energy minimization. In *Proceedings of the 2023 AAAI Fall Symposium on Integrating Cognitive Architectures and Generative Models* (AAAI Press)

Oscar J. Romero, A. S. A. T., John Zimmerman (2023). Synergistic integration of large language models and cognitive architectures for robust ai: An exploratory analysis. In *Proceedings of the 2023 AAAI Fall Symposium on Integrating Cognitive Architectures and Generative Models* (AAAI Press)

Park, J. S., O'Brien, J., Cai, C. J., Morris, M. R., Liang, P., and Bernstein, M. S. (2023). Generative agents: Interactive simulacra of human behavior. In *Proceedings of the 36th Annual ACM Symposium on User Interface Software and Technology*. 1–22

Xu, T., Singh, K., and Rajivan, P. (2022). Modeling phishing decision using instance based learning and natural language processing. In *HICSS*. 1–10

Zhou, L., Wu, X., Zhu, D., Cheng, M., Chen, S., Zhang, F., et al. (2023). Generative model-based testing on decision-making policies. *IEEE/ACM International Conference on Automated Software Engineering (ASE)*
